# Scientists’ warning on the global destruction of rock outcrop ecosystems

**DOI:** 10.1111/cobi.70316

**Published:** 2026-05-09

**Authors:** Luiza F. A. de Paula, Lucas N. Perillo, Gianluigi Ottaviani, Flávio F. Carmo, Mandar N. Datar, Katherine C. Enebeli‐Ekwutoziam, Bruno Fogliani, Stephen D. Hopper, Aboli Kulkarni, Hans Lambers, Rafael F. Magalhães, Frederico S. Neves, Jean‐François Ponge, Stefan Porembski, Bruno H. P. Rosado, Fernando M. G. Santos, Elycée Tindano, Bram Vanschoenwinkel, Aparna Watve, Fernando A. O. Silveira

**Affiliations:** ^1^ Departamento de Genética, Ecologia e Evolução Universidade Federal de Minas Gerais Belo Horizonte Brazil; ^2^ Research Institute on Terrestrial Ecosystems (IRET) National Research Council (CNR) Porano Italy; ^3^ National Biodiversity Future Center (NBFC) Palermo Italy; ^4^ Instituto Prístino Belo Horizonte Brazil; ^5^ Agharkar Research Institute Pune India; ^6^ Institut für Biologie und Umweltwissenschaften Carl von Ossietzky University of Oldenburg Oldenburg Germany; ^7^ ISEA University of New Caledonia Nouméa New Caledonia; ^8^ Albany Centre and School of Biological Sciences University of Western Australia Perth Western Australia Australia; ^9^ The Green Concept Pune India; ^10^ University of Western Australia Perth Western Australia Australia; ^11^ Departamento de Ciências Naturais Universidade Federal de São João del‐rei São João del rei Brazil; ^12^ Muséum National d'Histoire Naturelle, UMR 7179 MECADEV Brunoy France; ^13^ Institut für Biodiversitätsforschung i. Gr. Universität Rostock Rostock Germany; ^14^ Departamento de Ecologia, IBRAG Universidade Estadual do Rio de Janeiro Rio de Janeiro Brazil; ^15^ Université Thomas Sankara Saaba Burkina Faso; ^16^ Community Ecology Lab, bDIV Research Group, Department of Biology Vrije Universiteit Brussel (VUB) Brussels Belgium; ^17^ Center for Environmental Management University of the Free State Bloemfontein South Africa; ^18^ IUCN SSC Western Ghats Plant Specialist Group Pune India

**Keywords:** biodiversity hotspots, endemism, energy‐transition metals, inselbergs, mining, restoration, sky islands, species translocation, endemismo, inselbergs, islas celestes, metales de transición energética, minería, restauración, translocación de especies, zonas clave para la biodiversidad, 生物多样性热点, 特有性, 能源转型金属, 孤山, 采矿, 生态恢复, 天空岛, 物种迁移

## Abstract

Rock outcrops are geological formations that harbor a highly specialized biota adapted to harsh environmental conditions that differ from their surrounding landscapes. They are globally distributed, especially in old, highly weathered landscapes, and can function as habitat islands containing high levels of endemism and distinct evolutionary lineages. Despite their ecoevolutionary importance, rock outcrops remain largely overlooked in global conservation policy, ecosystem typologies, and biodiversity monitoring frameworks. Extractive activities directly remove the geological substrate that underpins these habitats, and climate change intensifies drought, heatwaves, and hydrological instability. Rising global demand for energy‐transition metals required for decarbonization is expected to further accelerate these pressures, particularly in biodiverse regions of the Global South where governance is weaker. Given that rock outcrop ecosystems are ecologically and evolutionarily significant, we devised a policy‐relevant conservation roadmap structured around prevention, mitigation, restoration, and governance issues. Priority actions include recognizing rock outcrops as unique ecosystems in global classifications and ecosystem typologies, integrating them into biodiversity monitoring frameworks, and improving the identification of key biodiversity areas and the International Union for Conservation of Nature (IUCN) Red List of Ecosystems assessments. We call for stronger environmental impact assessments, cumulative‐impact evaluations for mining and quarrying projects, and the designation of mining and quarrying exclusion sites of high irreplaceability. Coordinated international policy, improved ecological data, and meaningful engagement with local and Indigenous communities are essential to safeguard these ancient ecosystems before further, irreversible losses occur.

## Abstract (es)

Advertencia de los científicos sobre la destrucción global de los ecosistemas de afloramientos rocosos

**Resumen:**

Los afloramientos rocosos son formaciones geológicas que albergan una biota altamente especializada, adaptada a condiciones ambientales adversas que difieren de los paisajes circundantes. Se distribuyen por todo el mundo, especialmente en paisajes antiguos y muy erosionados, y pueden actuar como islas de hábitat con altos niveles de endemismo y linajes evolutivos distintivos. A pesar de su importancia ecoevolutiva, los afloramientos rocosos siguen siendo en gran medida ignorados en las políticas de conservación mundiales, las tipologías de ecosistemas y los marcos de seguimiento de la biodiversidad. Las actividades extractivas eliminan directamente el sustrato geológico que sustenta estos hábitats, y el cambio climático intensifica la sequía, las olas de calor y la inestabilidad hidrológica. Se prevé que el aumento de la demanda mundial de metales para la transición energética, necesarios para la descarbonización, acelere aún más estas presiones, especialmente en las regiones con gran biodiversidad del Sur Global, donde la gobernanza es más débil. Los ecosistemas de afloramientos rocosos revisten gran importancia desde el punto de vista ecológico y evolutivo. Por ello, hemos elaborado un protocolo para la conservación, relevante desde el punto de vista normativo, estructurada en torno a cuestiones de prevención, mitigación, restauración y gobernanza. Entre las medidas prioritarias se incluyen el reconocimiento de los afloramientos rocosos como ecosistemas únicos en las clasificaciones y tipologías de ecosistemas a nivel mundial, su integración en los marcos de seguimiento de la biodiversidad, y la mejora de la identificación de las áreas clave para la biodiversidad y de las evaluaciones de la Lista Roja de Ecosistemas de la Unión Internacional para la Conservación de la Naturaleza (UICN). Pedimos evaluaciones de impacto ambiental más rigurosas, evaluaciones del impacto acumulativo de los proyectos de minería y explotación de canteras, y la designación de zonas de exclusión para la minería y la explotación de canteras que sean irremplazables. Una política internacional coordinada, la mejora de los datos ecológicos y una participación significativa de las comunidades locales e indígenas son esenciales para salvaguardar estos ecosistemas ancestrales antes de que se produzcan más pérdidas irreversibles.

## 科学家对全球范围内岩石露头生态系统遭受破坏发出警告

**【摘要】** 岩石露头是一种地质构造, 其中栖息着高度特化的生物群落, 它们适应了不同于周围景观的严酷环境条件。这些地质构造分布于世界各地, 尤其常见于古老且风化程度高的景观中, 能够作为栖息地岛屿, 支持高特有性的物种及独特的演化谱系。尽管其具有重要的生态演化重要性, 但在全球保护政策、生态系统类型及生物多样性监测框架中, 它们仍很大程度上被忽视。采掘活动直接移除了支撑这些栖息地的地质基底, 而气候变化则加剧了干旱、热浪及水文不稳定性。全球对能源脱碳所需能源转型金属需求的增长, 预计将进一步加剧这些压力, 特别是在全球南方治理能力较弱而生物多样性丰富的地区。岩石露头生态系统在生态学和演化生物学上具有重要意义。因此, 本研究制定了一份以预防、减缓、恢复和治理问题为核心、具有政策指导意义的保护路线图。我们建议的优先行动包括:在全球分类体系和生态系统类型中将岩石露头认定为独特的生态系统;将其纳入生物多样性监测框架;以及改进关键生物多样性区域的识别和《国际自然保护联盟(IUCN)生态系统红色名录》的评估工作。我们呼吁加强环境影响评估, 对采矿和采石项目进行累积影响评估, 并将不可替代性高的地区划定为禁止采矿和采石的区域。在进一步出现不可逆的丧失之前, 制定协调一致的国际政策、增加生态数据, 并与当地和原住民社区开展实质性合作, 对于保护这些古老的生态系统至关重要。**【翻译:胡怡思;审校:聂永刚】**

## INTRODUCTION

The Scientists’ Warning to Humanity series (https://scientistswarning.org/about‐scientists‐warning/) has drawn attention to ongoing biodiversity and climate crises, emphasizing the urgent need for action. Unfortunately, rock outcrops and associated ecosystems (Table 1) are underrepresented in global conservation discussions and policy. These isolated geological features, also known as inselbergs *sensu lato*, include rock formations, such as *bornhardts*, *mesas*, cliffs, *koppies*, *nubbins*, and tower karst (Vanschoenwinkel et al., 2025), can be tens of millions of years old, and serve as refuges and refugia for biodiversity and specialized biota (Garnica‐Díaz et al., 2023; Keppel et al., 2012; Nyberg et al., 2025; Pillon et al., 2021; Seidou et al., 2025; Tibbett, 2015; Tindano et al., 2023). The neglect of rock outcrops in conservation planning, restoration schemes, and policy may have different explanations, such as generally small spatial extent, fragmented distribution, frequent classification within surrounding vegetation, and the misconception that rock outcrops are of low conservation value or are already degraded. Recent studies have raised concerns about the conservation status of rock outcrops. Such studies have focused primarily on specific taxa, biogeographic region, or type of impact. Here, we considered the ecological and evolutionary importance of rock outcrop biota in light of emerging threats, such as mining, quarrying, and climate change. We propose an integrated assessment of rock outcrops globally to provide policy guidelines for a roadmap to their long‐term conservation.

**TABLE 1:** Glossary of terms used in the conservation of rock outcrop ecosystems.

Term	Definition
Conservation psychology	Interdisciplinary field applying insights from psychology and behavioral sciences to understand and encourage human behaviors that support biodiversity conservation
Endemism	Condition in which a taxon is restricted to a specific geographic area; in our case, endemism refers to species that occur only on rock outcrops and are highly specialized to specific habitat conditions
Energy‐transition metal	Metal required for renewable energy technologies and low‐carbon infrastructure, including lithium, cobalt, nickel, copper, and other rare earth elements
Extractive activities	Industrial processes that remove natural resources from the earth, such as mining and quarrying
Granite–gneiss outcrop	Rock formation composed of granite or gneiss, often characterized by shallow soils, supporting unique and highly specialized plant communities
Inselberg (*sensu lato*)	Old, spatially isolated rock outcrop characterized by microhabitats that may be rare or absent in the surrounding matrix, supporting endemic and highly specialized biota
Irreplaceability	Metric describing the extent to which a site is essential for achieving biodiversity conservation goals because the species or ecosystems it hosts are rare or unique
Ironstone outcrop	Ferruginous rock formation rich in iron oxides supporting unique and highly specialized plant communities
Key biodiversity area (KBA)	Site identified, based on standardized global criteria of the International Union for Conservation of Nature, as contributing significantly to the persistence of biodiversity
Limestone outcrop (karst system)	Rock formation composed primarily of calcium carbonate, characterized by dissolution features, such as caves, sinkholes, and crevices, that create unique habitats supporting highly specialized plant communities
Mitigation hierarchy	Framework used in environmental management that prioritizes avoiding impacts, then minimizing them, restoring affected areas, and finally offsetting residual impacts
Microhabitat	Small‐scale habitat with environmental conditions and putative main ecological processes that differ from the surrounding area, often harboring unique and specialist species
Quartzite outcrop	Rock formation composed primarily of quartzite, often associated with nutrient‐poor substrates, supporting unique and highly specialized plant communities
International Union for Conservation of Nature Red List of Ecosystems	Global standard according to the International Union for Conservation of Nature for assessing risks to ecosystems, allowing the identification of common indicators to understand the level of risk that an ecosystem is facing
Refugium	Region (macrorefugium) or habitat (microrefugium) offering long‐term stable and predictable environmental conditions wherein components of biodiversity (often threatened rare species) may retreat to and persist under changing environments and can eventually recolonize the surroundings when conditions ameliorate; expected to operate as “biodiversity safe havens” over ecoevolutionary timescales (e.g., millennia), whereas refuges are short‐term ecological reservoirs (e.g., years)
Rock outcrop	Exposed bedrock formation that emerges above the surrounding soil or vegetation matrix, often creating isolated environmental conditions and supporting highly specialized species assemblages
Rock outcrop ecosystems	Ecosystems associated with exposed rock outcrop formations characterized by shallow soils, specialized biota, strong environmental filtering, and high levels of endemism
Serpentine outcrop	Ultramafic rock formation rich in iron, magnesium, and heavy metals, forming resource‐poor and highly toxic soils for many plants, supporting unique and highly specialized (e.g., hyperaccumulators) plant communities
Species turnover	Replacement of species from one location to another across space or time, contributing to patterns of beta diversity
Vernal pools or ephemeral rock pools	Temporary water bodies that accumulate in depressions on rock surfaces during rainy periods, supporting specialized aquatic organisms adapted to seasonal drying

## DISTRIBUTION AND ECOLOGICAL CONTEXT

Rock outcrops are hard, inorganic, geological features of various forms and sizes that are typically composed of granite, gneiss, quartzite, sandstone, basalt, ironstone, serpentinite, or limestone, among others (Vanschoenwinkel et al., 2025). Beyond their physical presence in the landscape, they often function as habitat islands that support specialized and frequently endemic biota, act as refugia during environmental change, and influence surrounding ecosystems through effects on microclimate, hydrology, and species dispersal (Fitzsimons & Michael, 2017; Vanschoenwinkel et al., 2025). Rock outcrops comprise open‐canopy vegetation and associated biota established on these geological features, typically characterized by spatial isolation, shallow soils, and strong environmental filtering (Bondi et al., 2024; de Paula et al., 2015; Hulshof & Spasojevic, 2020; Ottaviani & Keppel, 2018; Poot et al., 2012; Vanschoenwinkel et al., 2025).

**FIGURE 1:**
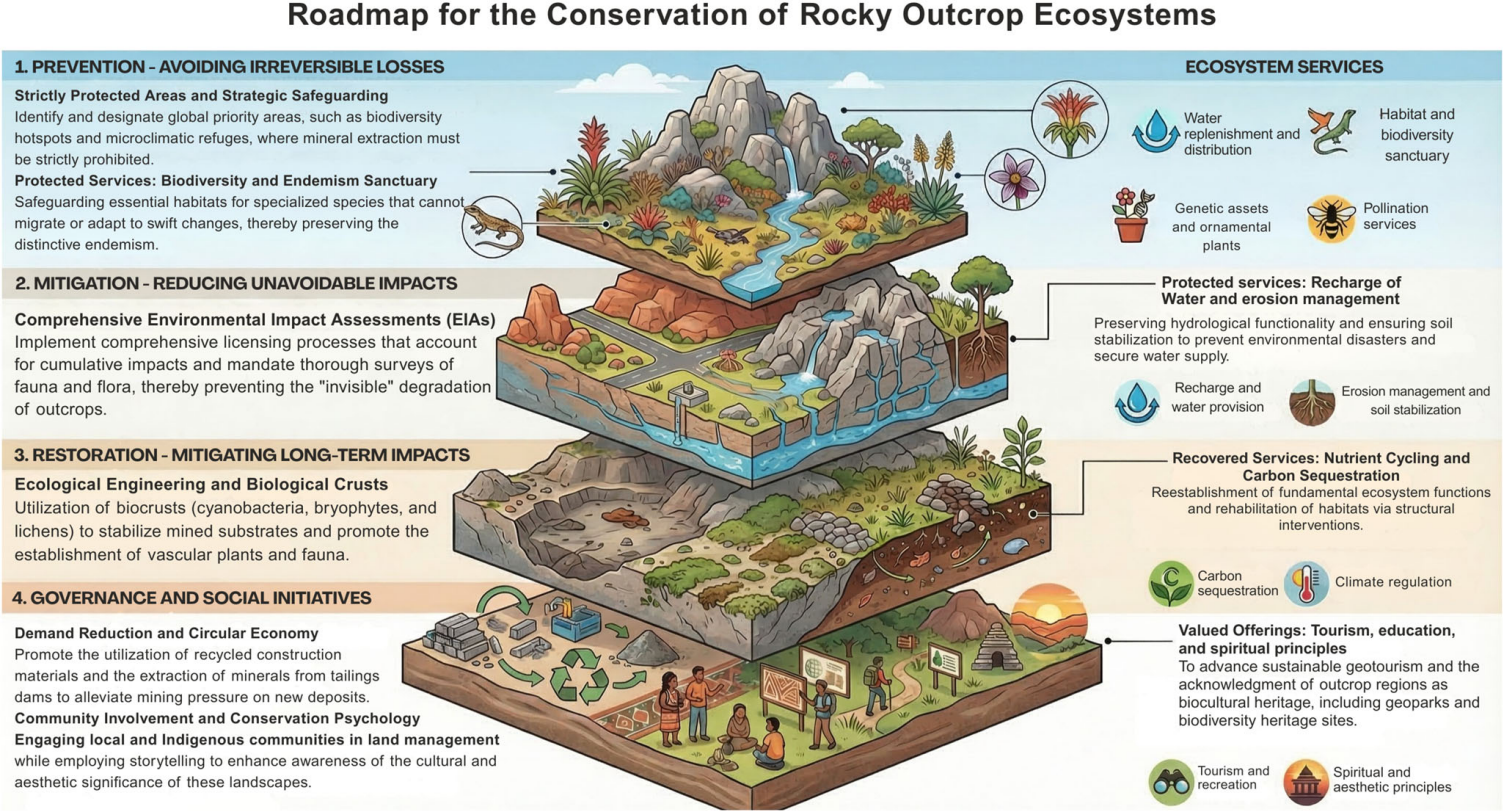
Roadmap for the conservation of rocky outcrop ecosystems structured around the key dimensions of prevention, mitigation, restoration, and governance to guide policy‐relevant conservation actions. Major ecosystem services and values of rocky outcrops are categorized as provisioning, regulating, supporting, and cultural.

These rocky systems are globally distributed but are especially common in ancient and highly weathered landscapes; famous examples are the Mt Roraima in northern South America, the Sugarloaf Mountain in Brazil, and Porongurup in Australia, where they host distinctive vegetation adapted to harsh oligotrophic conditions (Hopper, Lambers, et al., 2021; Vanschoenwinkel et al., 2025). Major formations include sandstone and quartzite outcrops in northern South America (e.g., *tepuis*), Brazil (e.g., *campo rupestre*), and Southern Africa; sandstone cliffs in West Africa; granite outcrops in Brazil (e.g., *campo de altitude*), India, Africa (including Madagascar), and southwestern Australia; lateritic plateaus and basalt cliffs in India (e.g., Western Ghats); and tower karsts in China. Rock outcrops harbor unique biodiversity and contribute to soil formation through rock weathering, runoff‐driven sediment deposition, and vegetation established on shallow soils and provide ecosystem services to millions of people (Abimbola & Ojo, 2015; Oliveira, Figueredo, et al., 2025). Rock outcrops provide provisioning, regulating, supporting, and cultural services (Millennium Ecosystem Assessment, 2005), including water recharge, plant resources, carbon storage, erosion control, microclimatic buffering, habitat provision, pollination, and cultural values, such as tourism, recreation, spiritual, and aesthetic appreciation (Figure 1; Appendix S1).

Rock outcrop biodiversity is shaped by local climatic (Brendonck et al., 2015; Clegg et al., 2025; de Paula et al., 2021) and unique microhabitat conditions associated with the exposed rocky terrain, such as high UV irradiance, shallow soils, water deficits, nutrient limitation, and variable exposure to disturbances, such as fires and herbivores (Vanschoenwinkel et al., 2025). Due to the presence of unique microhabitats (e.g., boulders, seeps, rock pools, gorges with moist microclimates, exposed rock slabs with arid microclimates) that are absent or rare in the surrounding landscape matrix, rock outcrops can harbor high levels of biodiversity, rare and outcrop‐endemic taxa restricted to one or few outcrops with relictual distributions (Akinyede et al., 2017; Oswald et al., 2025; Vanschoenwinkel et al., 2025), and often substantial species turnover across outcrops (de Paula et al., 2021; Hopper, Fiedler, et al., 2021; Perillo et al., 2021). Rock outcrops may function both as sites of recent colonization by plant and animal lineages and as centers of diversification of ancient autochthonous clades, thus generating a mosaic of distinct evolutionary trajectories (Johnson et al., 2022; Merckx et al., 2015; Salerno et al., 2012).

Given their old age, isolation, and the presence of distinct microhabitats, the biota of rock outcrops is often highly specialized (Vanschoenwinkel et al., 2025). Many outcrop species are characterized by life‐history strategies and trait combinations that may be explained by physiological trade‐offs, often different from those found in the surrounding landscape matrix (Ititiaty et al., 2020; Ottaviani & Keppel, 2018; Marks et al., 2025; Matos et al., 2021). The distribution of these traits often has implications for conservation and restoration. For example, many outcrop‐endemic species exhibit limited dispersal abilities but enhanced local persistence strategies, with offspring establishing close to parent plants, which reduces their ability to shift distributions and track suitable climatic conditions under rapid environmental change (Cheplick, 2022; Corlett & Tomlinson, 2020). Rock outcrop plants have evolved a suite of alternative strategies to persist under chronic water and nutrient scarcity, including desiccation tolerance (Bondi et al., 2024; Seidou et al., 2025), the formation of monocot mats (de Paula et al., 2021), specialized belowground organs, traits that enhance resource acquisition (e.g., phosphorus‐mining root systems [Jaffré, 2023; Lambers et al., 2010]), efficient resource use and storage, resprouting after disturbance (Ottaviani et al., 2022; Poot et al., 2012), and increased allocation to vegetative reproduction (Centenaro et al., 2023). Reduced allocation to sexual reproduction (probably linked to resource‐poor soils limiting the acquisition of essential resources to support it) may also constrain long‐term population viability (Fujita et al., 2014) through the Muller's ratchet effect.

Invertebrates of outcrops include specialists and opportunistic species that often exploit resources with time‐limited availability, such as the short growing or flowering season of plants or the inundations of temporary ponds (Jocqué et al., 2010; Neves et al., 2021). In Brazil, insect assemblages associated with rock outcrops show strong spatial and temporal variation across multiple groups, with turnover exceeding 60% for ants, bees, wasps, butterflies, and plant–pollinator networks (Neves et al., 2025; Perillo et al., 2023). High frequencies of singletons, doubletons, and tripletons further suggest that these communities include a substantial proportion of rare species (Perillo et al., 2021). Ironstone outcrops harbor a high richness of troglobitic invertebrates (cave specialists) and stygobites (groundwater specialists), including arachnids, hemipterans, hexapods, and myriapods (Zeppelini et al., 2022). Many species are known only from their type localities, underscoring the critical importance of conserving these habitats (Ferreira et al., 2019; Gibson et al., 2015). Several rock outcrops harbor pools and temporary streams hosting specialist macro‐ and microcrustaceans, such as fairy, clam, and tadpole shrimps, seed shrimps, copepods, and water fleas (Jocqué et al., 2010). These crustaceans are typically characterized by rapid growth and reproduction, and many have dormant life stages like annual plants (Pinceel et al., 2021). Those with dormant stages are often poor dispersers, while flying adults of aquatic insects (typically lacking dormant stages) rely on effective colonization (Vanschoenwinkel et al., 2013).

Amphibians associated with rock outcrops in arid regions are often explosive breeders that emerge and breed only after abundant rains and aestivate in summer, and some have camouflage to blend in with the rocky terrain (Reniers et al., 2015). In the Neotropics, several outcrop frogs breed in large tank bromeliads that include outcrop endemic species, often exhibiting strong behavioral and life‐history specializations that often constrain their colonization ability (Oswald et al., 2025). Similar patterns of restriction and specialization occur in reptiles that depend on rocky substrates for shelter, thermoregulation, and predator avoidance (Lajmi et al., 2020). More mobile organisms like birds and mammals are rarely restricted to individual outcrops and outcrop habitats in general. However, some cave‐dwelling bats and cliff‐breeding birds may show a strong preference for rock outcrops and may be reliant on them for successful reproduction. These examples illustrate how rock outcrops provide critical habitats for specialized vertebrates with strong site fidelity (Fitzsimons & Michael, 2017; Jithin et al., 2023; Piñeiro et al., 2021).

## HISTORICAL AND EMERGING THREATS TO ROCK OUTCROP ECOSYSTEMS

Rock outcrops have historically been less prone to conversion to agriculture or deforestation than the surrounding matrix due to their topography and because their shallow soils do not represent profitable farmland. Nonetheless, many outcrops are subject to grazing and vegetation removal (e.g., for collection of firewood or ornamental plants) and face additional threats, such as soil compaction, erosion, biological invasion, windfarm construction, afforestation, nutrient enrichment, altered fire regimes, and urbanization (Fitzsimons & Michael, 2017; Monteiro et al., 2018). The severity of these threats varies geographically and depends on topography, socioeconomic contexts, and governance structures. Although rock outcrops are most commonly assessed as threatened under criterion B (restricted geographic range) of the International Union for Conservation of Nature (IUCN) Red List of Ecosystems (RLE) (Bland et al., 2017), according to IUCN's criteria, degradation processes affecting their ecological function are often poorly documented due to limited long‐term data, suggesting that cumulative impacts may have been underestimated.

Mining, quarrying, and climate change emerge as the most important threats, which, in combination, increase vulnerability in these systems by intensifying thermal stress, drought exposure, and hydrological instability (Carmo et al., 2020; Fernandes et al., 2018; Salles et al., 2019; Souza‐Filho et al., 2019; Tibbet, 2015). These three drivers represent globally widespread pressures that directly affect rock outcrop ecosystems either through physical removal of the substrate (mining and quarrying) or through large‐scale environmental change that amplifies ecological stress (climate change). Together, they capture direct and indirect mechanisms of ecosystem degradation reported across multiple outcrop systems worldwide (Vanschoenwinkel et al., 2025).

Because rock outcrops represent exposed lithologies that are sometimes mineral rich, they can be attractive targets for mining industries. For instance, ferricretes and banded iron formations associated with ironstone outcrops are intensively mined in Brazil (Carmo et al., 2020) and Australia (Gibson et al., 2010, 2015), often coinciding with centers of plant endemism. However, beyond the intense impact of mining of iron ore, the projected surge in demand for energy‐transition metals critical for decarbonization and clean energy technologies further intensifies threats. Demands for lithium, cobalt, nickel, copper, and rare earth elements required for batteries and renewable energy infrastructure is expected to substantially increase mining activity by nearly five times by 2050 globally (Hund et al., 2020; Nijnens et al., 2023; Sonter et al., 2023; Watari et al., 2019). In turn, quarrying represents a related but distinct pressure. Lithologies commonly associated with rock outcrops (e.g., granite, gneiss, laterite, and limestone) are increasingly extracted to support construction and infrastructure expansion, particularly in rapidly urbanizing regions. For example, lateritic plateaus of the Western Ghats in India have been quarried for centuries for building materials (Kaur et al., 2023), and granite and gneiss outcrops are widely exploited for construction stone in parts of Africa, Brazil, and India (Porembski et al., 2016). Similarly, limestone and karst formations worldwide are heavily quarried for cement production and industrial materials (Bystriakova et al., 2019). Unlike large‐scale mining for metals, quarrying is often spatially fragmented but widespread, leading to progressive physical removal and fragmentation of outcrop habitats.

High‐priority areas for mining and quarrying frequently overlap with biodiversity hotspots (Lèbre et al., 2020), intensifying tensions between decarbonization goals and biodiversity commitments under the Kunming–Montreal Global Biodiversity Framework (2022) adopted under the Convention on Biological Diversity. These pressures are particularly acute in the Global South, where many biodiverse outcrops occur, and regulatory safeguards may be weaker, amplifying biodiversity loss and environmental injustice (Sonter et al., 2023; Stacciarini & Gonçalves, 2025; Urban, 2024).

Climate change, in turn, is another relevant activity that further impacts outcrops. Evidence from multiple regions documents climate‐related impacts on outcrop vegetation, including shifts in species composition, altered fire regimes, and increased mortality during extreme heat and drought events (e.g., Fitzsimons & Michael, 2017; Nyberg et al., 2025). A climate simulation study showed that a substantial fraction of rock outcrop fairy shrimp populations will be vulnerable to extinction under future climate scenarios (Pinceel et al., 2018). However, vulnerability assessments that explicitly integrate species sensitivity (degree to which species performance and persistence rely on climate variables likely to change), exposure (magnitude and rate of climate change in occupied habitats), and adaptive capacity (ability to cope by persisting in situ, shifting to microhabitats, or migrating) (Dawson et al., 2011) remain scarce for rock outcrop floras (Bondi et al., 2024; Pinceel et al., 2018). Because many rock outcrop specialist plants exhibit narrow ecological niches, high habitat fidelity, and limited dispersal, their capacity to track shifting climatic envelopes is deeply constrained (Corlett & Tomlinson, 2020).

## CALL FOR ACTION TO PROTECT ROCK OUTCROPS GLOBALLY

Consequences of inaction to protect rock outcrops include irreversible loss of unique species and of functional and evolutionary diversity, erosion of ecosystem services (Figure 1; Appendix S1), large‐scale landscape degradation, and irreparable socioeconomic damage. Protection of rock outcrops must be the primary strategy considering that full ecological restoration is not feasible after quarrying and open‐pit mining or when the local conditions no longer support the biota. In the case of other threats, such as overgrazing or unmanaged fires, restoration strategies are being developed, but restoration is extremely slow and often only partial recovery can be achieved (Arruda et al., 2023; Thorpe & Watve, 2015). Improving the protection of outcrops involves different challenges. Designating rock outcrops as conservation sites may generate limited stakeholder resistance when they are on cheap land with limited opportunities for economic development but difficult when there are opportunities for extractive profits in the forms of mining or quarrying that generate conflicting interests for different stakeholders (Losekann & Milanez, 2023).

Relatively small areas that are ecologically distinct from their surrounding matrix have largely been overlooked in global assessments (Wintle et al., 2019), and this finding is likely true of rock outcrops as well. For example, the IUCN Global Ecosystem Typology (Keith et al., 2020) typically treats rock outcrops as part of the surrounding landscape matrix, which ensures that their ecological distinctness is unaccounted for. Consequently, rock outcrops are nearly invisible in monitoring frameworks, such as those underpinning the Global Biodiversity Framework. Although rock outcrops frequently meet the criteria for designation as key biodiversity areas (KBAs) (IUCN, 2016), especially under the criteria concerning geographically restricted and threatened biodiversity, they are often omitted from KBA identification and mapping.

Mapping and assessing outcrops under the KBA framework could provide a structured pathway for improved recognition and prioritization in national and subnational conservation planning. In many regions, however, individual outcrops are unlikely to qualify as standalone KBAs. Instead, clusters or archipelagos of outcrops that collectively sustain high levels of endemism, species turnover, and ecological complementarity may represent more appropriate assessment units. Such efforts, although not conferring automatic legal protection, can strengthen the scientific basis for policy decisions and resource allocation. Incorporating zonation of surrounding buffers (Buschke et al., 2020), similar to wetland regulatory frameworks, may further enhance management effectiveness. In parallel, RLE assessments at finer scales (levels 5 and 6) are critical to capture the restricted extent, functional fragility, and vulnerability to cumulative losses of rock outcrop ecosystems. Greater alignment between KBA and RLE approaches could therefore improve the visibility of these systems within conservation planning processes and help reduce incremental losses driven by fragmented licensing and small‐scale extraction.

Although IUCN Red List assessments are crucial for connecting scientific data to public policy, only 18% of vascular plants had been assessed by IUCN (Bachman et al., 2024), and only 1.4% of invertebrates have been assessed (Bánki et al., 2025), highlighting the need for greater efforts to meet international biodiversity targets. Most assessed species are woody perennials and useful plants, whereas single‐country endemic species remain underrepresented (Nic Lughadha et al., 2020), producing a critical gap for species associated with rock outcrops, most of which lack official conservation assessment (Nyberg et al., 2025).

## ROADMAP TO ROCK OUTCROP ECOSYSTEM CONSERVATION

We advocate for an integrated approach to prevent habitat loss and degradation and to reduce extinction risk of rare and endemic species, from local‐scale management actions to the recognition of large‐scale ecological and evolutionary processes. Beyond species‐level assessments and high‐level instruments, such as the IUCN Red List, mitigating impacts, particularly those associated with mining and quarrying, on rock outcrops requires a coordinated set of actions. We organized these actions into a policy‐relevant hierarchical conservation roadmap structured around prevention, mitigation, and restoration (Arlidge et al., 2018) and cross‐cutting societal and governance measures. Where impacts cannot be fully avoided, operational mitigation must follow the mitigation hierarchy, prioritizing avoidance, then minimization, and, only as a last resort, offsetting residual impacts (Arlidge et al., 2018).

### Preventing irreversible loss

Preventing impacts is the highest priority for rock outcrop ecosystems, given their geological specificity, high irreplaceability, and extremely limited potential for recovery once degraded. This includes designation of mining and quarrying exclusion sites where extraction is prohibited. Strategic planning in mining and quarrying must explicitly identify critical conservation priorities for rock outcrop endemic species, highly threatened or unique ecosystems, and World Heritage Sites and set these aside from development. International frameworks led by the International Finance Corporation, IUCN, and the International Council on Mining and Metals recognize critical habitats as areas subject to stringent conditional requirements, including demonstration of no viable alternatives, avoidance of measurable adverse impacts, and achievement of net gains where development proceeds (PS06 IFC, 2012; CSBI, 2015; Torres et al., 2024). Unfortunately, these principles remain inconsistently applied to rock outcrops.

Prevention also includes identifying global priority sites for new protected areas based on biodiversity hotspots, ecosystem services, irreplaceability, and anthropogenic impact vulnerability to ensure that rock outcrops are explicitly considered rather than aggregated within surrounding vegetation types. In the context of climate change, this prioritization should explicitly incorporate the identification and protection of microclimatic refugia with spatial ecological and microclimatic modeling approaches, maintenance of topographic and substrate heterogeneity, and safeguarding of connectivity among outcrops (e.g., preserving the surrounding matrix) across elevational and climatic gradients, thereby enhancing in situ persistence of species with limited dispersal capacity and narrow climatic tolerances (Corlett & Tomlinson, 2020).

### Mitigation of unavoidable impacts

Fragmented environmental licensing should be replaced by integrated assessments that consider cumulative impacts on entire outcrop systems. In critical habitats, mitigation strategies are required to achieve net gain for the biodiversity values that justified their designation. For rock outcrops, net gain cannot be interpreted as replacing degraded outcrops. Instead, it may include demonstrable improvements in the condition, extent, and long‐term security of remaining outcrops in the same ecological and biogeographic context combined with legally binding protection and management of set‐aside areas adjacent to or ecologically connected with affected sites.

Robust environmental impact assessments (EIAs) are essential. With such assessments, governments and practitioners adopt evidence‐based and risk‐based approaches, apply the precautionary principle when dealing with data‐deficient, rare, or threatened species, and explicitly account for cumulative impacts on the same critical habitats. Several studies have documented that insufficiently robust EIAs associated with quarrying, mining, and infrastructure development have contributed to the degradation of rock outcrop ecosystems in different regions of the world (e.g., Pimenta & Fonseca, 2021). For example, in southeastern Brazil, granite–gneiss outcrops are heavily affected by the ornamental stone industry, which generates approximately USD 1 billion annually and accounts for about 76% of Brazil's exports in this sector, concentrated in only 0.54% of the national territory (Francisco et al., 2023). The EIAs for these extractive activities often exclude mandatory fauna and flora surveys, likely because the vegetation is not classified as forested, leading to limited ecological information on these rock outcrops (Couto et al., 2019). Similar challenges have been documented in Australia, where mining and quarrying on banded ironstone and granite outcrops have proceeded despite the high levels of plant endemism and ecological specialization in these systems, highlighting how inadequate baseline ecological data in impact assessments can contribute to the decline of specialized outcrop ecosystems (Gibson et al., 2010). Where uncertainty is high due to lack of scientific evidence, adequate and funded research should precede approval, and the feasibility of effective management and restoration must be demonstrated.

### Addressing long‐term impacts with restoration

Given the long formation times and low resilience of rock outcrop vegetation (Arruda et al., 2023), restoration options are limited but increasingly necessary where impacts have already occurred. Developing approaches to enhance ecological restoration includes improving techniques for substrate stabilization, soil development, and species reestablishment, as well as anticipating restoration needs to reduce time‐lag uncertainties associated with biodiversity offsets (Cowan et al., 2020; Fernandes et al., 2023; Julien et al., 2022). Biological soil crusts (biocrusts), composed of cyanobacteria, bryophytes, algae, and lichens, are promising tools for restoring mining‐affected substrates. These pioneer communities stabilize soils, retain moisture, fix carbon and nitrogen, and facilitate vascular plant establishment, thereby supporting early ecosystem recovery on degraded substrates, such as mining tailings and exposed rock surfaces (Oliveira, Santos, et al., 2025). Recent restoration approaches also explore scalable inoculation techniques, including the encapsulation of biocrust organisms in alginate beads, which may improve their establishment and propagation in restoration contexts (Oliveira, Santos, et al., 2025). Because many rock outcrop plants are slow growing, habitat specialists, and dispersal limited, recovery cannot be assumed to be rapid, making long‐term monitoring and adaptive management essential (Vanschoenwinkel et al., 2025).

Structural interventions can support species recovery if they effectively recreate fine‐scale microhabitat conditions for endemic vertebrates (Alvarez & Guida‐Johnson, 2019). Artificial habitat reconstruction has been proposed as a refuge for outcrop endemic marsupials, but its effectiveness in supporting long‐term population while preventing establishment of invasive species remains to be assessed (Cowan et al., 2023). Where translocation is considered, global standards for mitigation translocation, the deliberate relocation of organisms from affected sites to alternative habitats, should be applied to minimize risks to source and recipient populations and to ensure long‐term monitoring and accountability (Doyle et al., 2023). In any case, restoration and offsets must remain a last resort within the mitigation hierarchy because restored sites and recreated structures rarely substitute for the geological and evolutionary integrity of intact rock outcrops.

### Governance and societal actions

Additional actions are required to reduce pressures at broader scales. These include reducing demand for energy‐transition metals and other rock‐derived materials through technological and societal changes, such as the use of materials from construction and demolition waste and the recovery of minerals from mining tailings dams (Torres et al., 2024). Establishing international regulatory policies to limit the trade of commodities whose extraction disproportionately threatens rock outcrop endemic species is also critical (e.g., Giulietti et al., 2019).

Further, preventing and reverting afforestation on naturally open rock outcrops is necessary in regions where tree planting for carbon sequestration or commercial forestry degrades endemic‐rich open ecosystems (Veldman et al., 2015). This requires the use of spatial planning tools, ecosystem‐specific definitions in restoration policy, and adaptive management approaches that recognize rock outcrops as valuable and irreplaceable open‐canopy ecosystems.

Finally, increasing awareness of the ecological and cultural importance of rock outcrops through evidence‐based conservation psychology approaches (application of behavioral science to encourage proenvironmental attitudes, Table 1) and other social science tools, such as framing, storytelling, and marketing, can strengthen public engagement and help build the societal support needed to influence governance, policy decisions, and corporate accountability relevant to their protection (Bennett et al., 2017).

For example, in the Western Ghats, participatory survey of biocultural values of lateritic plateaus has led to the designation of 11 biodiversity heritage sites and two conservation reserves (Chandran et al., 2012; Watve & Chavan, 2020) under the national laws. Meaningful involvement of local and Indigenous communities in conservation planning and decision‐making is essential to ensure equitable governance and reduce the risk of disproportionate social and environmental burdens associated with extractive activities (Kafu‐Quvane & Mlaba, 2024; Lullfitz et al., 2021). Where communities are excluded from decision‐making processes, extractive developments tend to exacerbate socioecological inequities and undermine long‐term conservation outcomes.

## CONCLUSIONS

Rock outcrops host ancient ecosystems characterized by high beta diversity and relatively high levels of endemism that are often impossible to restore after degradation or destruction. The oldest, largest, and those in megabiodiverse regions stand out as veritable treasures of biological diversity with unique genetic lineages and a rich reservoir of ecological and evolutionary diversity. Preventing losses requires immediate designation of mining and quarrying exclusion sites, explicit inclusion of rock outcrops in ecosystem typologies, tailored biodiversity monitoring frameworks, and rigorous application of the mitigation hierarchy. Investment in restoration science, climate adaptation, and evidence‐based translocation standards must be planned in advance rather than respond to after collapse. Without coordinated international policy, accountability, and meaningful engagement with local and Indigenous communities, rock outcrop ecosystems will continue to disappear without documentation. Recognizing and acting on their distinctiveness is an urgent conservation priority.

## AUTHOR CONTRIBUTIONS

Luiza F. A. de Paula, Lucas N. Perillo, and Fernando A. O. Silveira conceived the ideas and wrote the first draft of the manuscript with significant input from all coauthors.

## Supporting information



Supporting Information

## References

[cobi70316-bib-0001] Abimbola, F. A. , & Ojo, O. J. (2015). The hydrological significance of inselbergs in the savannah region of Nigeria. Journal of Applied Sciences and Environmental Management, 19(2), 355–362.

[cobi70316-bib-0002] Akinyede, J. O. , Ekanade, O. , & Oyebanji, O. (2017). Conservation of inselbergs in Nigeria: Challenges and opportunities. Nigerian Journal of Ecology, 21(1), 12–20.

[cobi70316-bib-0003] Alvarez, L. M. , & Guida‐Johnson, B. (2019). Habitat restoration for endemic lizards in an oilfield in Payunia, Argentina. Ecological Restoration, 37, 217–221.

[cobi70316-bib-0004] Arlidge, W. N. , Bull, J. W. , Addison, P. F. , Burgass, M. J. , Gianuca, D. , Gorham, T. M. , Jacob, C. , Shumway, N. , Sinclair, S. P. , Watson, J. E. M. , Wilcox, C. , & Milner‐Gulland, E. J. (2018). A global mitigation hierarchy for nature conservation. Bioscience, 68(5), 336–347. 10.1093/biosci/biy029 PMC5925785 29731513

[cobi70316-bib-0005] Arruda, A. J. , Medeiros, N. F. , Fiorini, C. F. , Ordóñez‐Parra, C. A. , Dayrell, R. L. C. , Messeder, J. V. S. , Zanetti, M. , Wardil, M. V. , Paiva, D. C. , Kozovits, A. R. , Buisson, E. , Le Stradic, S. , & Silveira, F. A. O. (2023). Ten principles for restoring campo rupestre, a threatened tropical, megadiverse, nutrient‐impoverished montane grassland. Restoration Ecology, 31(7), Article e13924. 10.1111/rec.13924

[cobi70316-bib-0006] Bachman, S. P. , Brown, M. J. M. , Leão, T. C. C. , Nic Lughadha, E. , & Walker, B. E. (2024). Extinction risk predictions for the world's flowering plants to support their conservation. New Phytologist, 242(2), 797–808. 10.1111/nph.19592 38437880

[cobi70316-bib-0007] Bánki, O. , Roskov, Y. , Döring, M. , Ower, G. , Hernández Robles, D. R. , Plata Corredor, C. A. , Stjernegaard Jeppesen, T. , Örn, A. , Pape, T. , Hobern, D. , Garnett, S. , Little, H. , DeWalt, R. E. , Ma, K. , Miller, J. , Orrell, T. , Aalbu, R. , Abbott, J. , Adlard, R. , … World Flora Online . (2025). Catalogue of Life (Version 2025‐02‐13). Catalogue of Life. 10.48580/dgnfb

[cobi70316-bib-0008] Bennett, N. J. , Roth, R. , Klain, S. C. , Chan, K. , Christie, P. , Clark, D. A. , Cullman, G. , Curran, D. , Durbin, T. J. , Epstein, G. , Greenberg, A. , Nelson, M. P. , Sandlos, J. , Stedman, R. , Teel, T. L. , Thomas, R. , Veríssimo, D. , & Wyborn, C. (2017). Conservation social science: Understanding and integrating human dimensions to improve conservation. Biological Conservation, 205, 93–108. 10.1016/j.biocon.2016.10.006

[cobi70316-bib-0009] Bland, L. M. , Keith, D. A. , Miller, R. M. , Murray, N. J. , & Rodríguez, J. P. (Eds.). (2017). Guidelines for the application of IUCN Red List of Ecosystems categories and criteria. Version 1.1. IUCN.

[cobi70316-bib-0010] Bondi, L. , Prado, B. , de Paula, L. F. , Rosado, B. H. , & Porembski, S. (2024). Rethinking the relationship between desiccation‐tolerant vascular plants and water deficit. Plant Ecology & Diversity, 17(1‐2), 1–19.

[cobi70316-bib-0011] Brendonck, L. M. , Jocqué, M. , Tuytens, K. , Timms, B. V. , & Vanschoenwinkel, B. (2015). Hydrological stability drives both local and regional diversity patterns in rock pool metacommunities. Oikos, 124, 741–749.

[cobi70316-bib-0012] Buschke, F. T. , Coetzer, C. , Pinceel, T. , Mehlomakhulu, Z. , Moreels, N. , Du Randt, L. , & Vanschoenwinkel, B. (2020). Mountains and rocky outcrops as ecological refuges in a high biodiversity working landscape. Biological Conservation, 250, Article 108759. 10.1016/j.biocon.2020.108759

[cobi70316-bib-0013] Bystriakova, N. , Alves De Melo, P. H. , Moat, J. , Lughadha, E. N. , & Monro, A. K. (2019). A preliminary evaluation of the karst flora of Brazil using collections data. Scientific Reports, 9(1), Article 17037. 10.1038/s41598-019-53104-6 PMC6863846 31745111

[cobi70316-bib-0014] Carmo, F. F. , Lanchotti, A. O. , & Kamino, L. H. (2020). Mining waste challenges: Environmental risks of gigatons of mud, dust and sediment in megadiverse regions in Brazil. Sustainability, 12, Article 8466.

[cobi70316-bib-0015] Centenaro, G. , Petraglia, A. , Carbognani, M. , Piotti, A. , Hudek, C. , Büntgen, U. , & Crivellaro, A. (2023). The oldest known clones of *Salix herbacea* growing in the Northern Apennines, Italy are at least 2000 years old. American Journal of Botany, 110(10), Article e16243. 10.1002/ajb2.16243 37755870

[cobi70316-bib-0016] Chandran, M. D. S. , Ramachandra, T. V. , Joshi, N. V. , Rao, G. R. , Mesta, P. N. , Balachandran, C. , & Dudani, S. N. (2012). Conservation reserve status to lateritic plateaus of coastal Uttara Kannada. Sahyadri Conservation Series 21 (ENVIS Technical Report 51). Environmental Information System (ENVIS).

[cobi70316-bib-0017] Cheplick, G. P. (2022). Philomatry in plants: Why do so many species have limited seed dispersal? American Journal of Botany, 109(1), 29–45. 10.1002/ajb2.1791 34679185

[cobi70316-bib-0018] Clegg, R. , Azevedo, L. , Martinez‐Ugarteche, M. T. , Neves, D. , Kidner, C. , Hind, D. J. N. , Antonelli, A. , Rowland, L. , & Pennington, R. T. (2025). Plant biogeography of rock outcrops in South American tropical lowlands. Frontiers of Biogeography, 18, Article e145659. 10.21425/fob.18.145659

[cobi70316-bib-0101] Convention on Biological Diversity (CBD) . (2022). Kunming‐Montreal Global Biodiversity Framework: Decision Adopted by the Conference of the Parties to the Convention on Biological Diversity, 19 December 2022, CBD/COP/DEC/15/4.

[cobi70316-bib-0019] Corlett, R. T. , & Tomlinson, K. W. (2020). Climate change and edaphic specialists: Irresistible force meets immovable object? Trends in Ecology & Evolution, 35(4), 367–376. 10.1016/j.tree.2019.12.007 31959419

[cobi70316-bib-0020] Couto, D. R. , Fontana, A. P. , Neto, R. , A, C. , Gomes, J. M. L. , Calazans, L. S. B. , Silva, H. L. , & Fraga, C. N. (2019). Angiospermas monocotiledôneas ameaçadas de extinção no estado do Espírito Santo. In C. N. Fraga , M. D. H. Formigoni , & F. G. Chaves (Eds.), Fauna e flora ameaçadas de extinção no estado do Espírito Santo (pp. 164–191). Instituto Nacional da Mata Atlântica.

[cobi70316-bib-0021] Cowan, M. A. , Callan, M. N. , Tippler, C. , & Nimmo, D. G. (2023). A technological advancement in artificial refuges for an endangered marsupial predator. Conservation Science and Practice, 5(8), Article e12981.

[cobi70316-bib-0022] Cowan, M. A. , Dunlop, J. A. , Turner, J. M. , Moore, H. A. , & Nimmo, D. G. (2020). Artificial refuges to combat habitat loss for an endangered marsupial predator: How do they measure up? Conservation Science and Practice, 2(6), Article e204.

[cobi70316-bib-0023] Cross Sector Biodiversity Initiative (CSBI) . (2015). A cross‐sector guide for implementing the mitigation hierarchy. The Biodiversity Consultancy on behalf of IPIECA.

[cobi70316-bib-0024] Dawson, T. P. , Jackson, S. T. , House, J. I. , Prentice, I. C. , & Mace, G. M. (2011). Beyond predictions: Biodiversity conservation in a changing climate. Science, 332(6025), 53–58. 10.1126/science.1200303 21454781

[cobi70316-bib-0025] de Paula, L. F. A. , Forzza, R. C. , Azevedo, L. O. , Bueno, M. L. , Solar, R. R. , Vanschoenwinkel, B. , & Porembski, S. (2021). Climatic control of mat vegetation communities on inselberg archipelagos in south‐eastern Brazil. Biological Journal of the Linnean Society, 133(2), 604–623.

[cobi70316-bib-0026] de Paula, L. F. A. , Negreiros, D. , Azevedo, L. O. , Fernandes, R. L. , Stehmann, J. R. , & Silveira, F. A. O. (2015). Functional ecology as a missing link for conservation of a resource‐limited flora in the Atlantic forest. Biodiversity and Conservation, 24(9), 2239–2253.

[cobi70316-bib-0027] Doyle, C. A. T. , Abeli, T. , Albrecht, M. A. , Bellis, J. , Colas, B. , Dalrymple, S. E. , Ensslin, A. , Espejo, J. , Erftemeijer, P. L. A. , Julien, M. , Lewandrowski, W. , Liu, H. , Moehrenschlager, A. , Ooi, M. K. J. , Reynolds, D. M. , Schatz, B. , Sild, M. , Wills, T. J. , & Papuga, G. (2023). Achieving conservation outcomes in plant mitigation translocations: The need for global standards. Plant Ecology, 224(9), 745–763.

[cobi70316-bib-0028] Fernandes, G. W. , Barbosa, N. P. U. , Alberton, B. , Barbieri, A. , Dirzo, R. , Goulart, F. F. , Guerra, T. J. , Morellato, L. P. C. , & Solar, R. R. C. (2018). The deadly route to collapse and the uncertain fate of Brazilian rupestrian grasslands. Biodiversity and Conservation, 27(10), 2587–2603.

[cobi70316-bib-0029] Fernandes, T. N. , Dos Santos, F. M. G. , Gontijo, F. D. , da Silva Filho, J. A. , Castilho, A. F. , & Sánchez, L. E. (2023). Mainstreaming flora conservation strategies into the mitigation hierarchy to strengthen environmental impact assessment. Environmental Management, 71(2), 483–493. 10.1007/s00267-022-01756-y PMC9892156 36459196

[cobi70316-bib-0030] Ferreira, R. L. , de Oliveira, M. P. A. , & Silva, M. S. (2019). Subterranean biodiversity in ferruginous landscapes. In O. Moldovan , L. Kováč , & S. Halse (Eds.), Cave ecology (Vol. 235, pp. 435–447). Springer. 10.1007/978-3-319-98852-8_21

[cobi70316-bib-0031] Fitzsimons, J. A. , & Michael, D. R. (2017). Rocky outcrops: A hard road in the conservation of critical habitats. Biological Conservation, 211, 36–44.

[cobi70316-bib-0032] Francisco, T. M. , Couto, D. R. , Moreira, M. M. , Fontana, A. P. , & De Fraga, C. N. (2023). Inselbergs from Brazilian Atlantic Forest: High biodiversity refuges of vascular epiphytes from Espírito Santo. Biodiversity and Conservation, 32, 2561–2584.

[cobi70316-bib-0033] Fujita, Y. , Venterink, H. O. , Van Bodegom, P. M. , Douma, J. C. , Heil, G. W. , Hölzel, N. , Jabłońska, E. , Kotowski, W. , Okruszko, T. , Pawlikowski, P. , De Ruiter, P. C. , & Wassen, M. J. (2014). Low investment in sexual reproduction threatens plants adapted to phosphorus limitation. Nature, 505(7481), 82–86. 10.1038/nature12733 24240278

[cobi70316-bib-0034] Garnica‐Díaz, C. , Berazaín Iturralde, R. , Cabrera, B. , Calderón‐Morales, E. , Felipe, F. L. , García, R. , Hechavarría, J. L. G. , Guimarães, A. F. , Medina, E. , Paul, A. L. D. , Rajakaruna, N. , Restrepo, C. , Siebert, S. J. , Van Den Berg, E. , Van Der Ent, A. , Velasquez, G. , & M Hulshof, C. (2023). Global plant ecology of tropical ultramafic ecosystems. The Botanical Review, 89(2), 115–157.

[cobi70316-bib-0035] Gibson, N. , Coates, D. , van Leeuwen, S. , & Yates, C. (2015). Hot, dry and ancient: Banded iron formations of Western Australia. In F. F. Carmo & L. H. Kamino (Eds.), Geossistemas ferruginosos do Brasil: Áreas prioritárias para conservação da diversidade geológica e biológica, patrimônio cultural e serviços ambientais (pp. 361–391). Instituto Prístino.

[cobi70316-bib-0036] Gibson, N. , Yates, C. , & Dillon, R. (2010). Plant communities of the ironstone ranges of South Western Australia: Hotspots for plant diversity and mineral deposits. Biodiversity and Conservation, 19(14), 3951–3962.

[cobi70316-bib-0037] Giulietti, A. M. , Giannini, T. C. , Mota, N. F. , Watanabe, M. T. , Viana, P. L. , Pastore, M. , Silva, U. C. S. , Siqueira, M. F. , Pirani, J. R. , Lima, H. C. , Pereira, J. B. S. , Brito, R. M. , Harley, R. M. , Siqueira, J. O. , & Zappi, D. C. (2019). Edaphic endemism in the Amazon: Vascular plants of the canga of Carajás, Brazil. The Botanical Review, 85(4), 357–383.

[cobi70316-bib-0038] Hopper, S. D. , Fiedler, P. L. , & Yates, C. J. (2021). Inselberg floristics exemplify coast to inland OCBIL transition in a global biodiversity hotspot. Biological Journal of the Linnean Society, 133, 624–644.

[cobi70316-bib-0039] Hopper, S. D. , Lambers, H. , Silveira, F. A. O. , & Fiedler, P. L. (2021). OCBIL theory examined: Reassessing evolution, ecology and conservation in the world's ancient, climatically buffered and infertile landscapes. Biological Journal of the Linnean Society, 133(2), 266–296.

[cobi70316-bib-0040] Hulshof, C. M. , & Spasojevic, M. J. (2020). The edaphic control of plant diversity. Global Ecology and Biogeography, 29(10), 1634–1650.

[cobi70316-bib-0041] Hund, K. , La Porta, D. , Fabregas, T. , Laing, T. , & Drexhage, J. (2020). Minerals for climate action: The mineral intensity of the clean energy transition. World Bank Group, Climate‐Smart Mining Facility.

[cobi70316-bib-0102] International Finance Corporation (IFC) . (2012). Performance Standards on Environmental and Social Sustainability: Performance Standard 6Biodiversity Conservation and Sustainable Management of Living Natural Resources . World Bank Group.

[cobi70316-bib-0042] International Union for Conservation of Nature (IUCN) . (2016). A global standard for the identification of Key Biodiversity Areas: Version 1.0 . Author.

[cobi70316-bib-0043] Ititiaty, Y. , Brescia, F. , Bordez, L. , Gensous, S. , McCoy, S. , & Fogliani, B. (2020). Life traits of ultramafic plant taxa from the Goro plateau in the tropical hotspot of New Caledonia. Restoration Ecology, 28(6), 1505–1516. 10.1111/rec.13248

[cobi70316-bib-0044] Jaffré, T. (2023). Plant mineral nutrition on ultramafic rocks of New Caledonia. Botany Letters, 170(3), 375–411. 10.1080/23818107.2022.2080112

[cobi70316-bib-0045] Jithin, V. , Rane, M. , Watve, A. , Giri, V. B. , & Naniwadekar, R. (2023). Between a rock and a hard place: Comparing rock‐dwelling animal prevalence across abandoned paddy, orchards, and rock outcrops in a biodiversity hotspot. Global Ecology and Conservation, 46, Article e02582. 10.1016/j.gecco.2023.e02582

[cobi70316-bib-0046] Jocqué, M. , Vanschoenwinkel, B. , & Brendonck, L. (2010). Freshwater rock pools: A review of habitat characteristics, faunal diversity and conservation value. Freshwater Biology, 55(8), 1587–1602. 10.1111/j.1365-2427.2010.02402.x

[cobi70316-bib-0047] Johnson, J. , Loria, S. F. , Joseph, M. M. , & Harms, D. (2022). Biogeographical and diversification analyses of Indian pseudoscorpions reveal the Western Ghats as museums of ancient biodiversity. Molecular Phylogenetics and Evolution, 175, Article 107495. 10.1016/j.ympev.2022.107495 35569808

[cobi70316-bib-0048] Julien, M. , Colas, B. , Muller, S. , & Schatz, B. (2022). Quality assessment of mitigation translocation protocols for protected plants in France. Journal of Environmental Management, 302, Article 114064. 10.1016/j.jenvman.2021.114064 34800770

[cobi70316-bib-0049] Kafu‐Quvane, B. , & Mlaba, S. (2024). Assessing the impact of quarrying as an environmental ethic crisis: A case study of limestone mining in a rural community. International Journal of Environmental Research and Public Health, 21(4), Article 458. 10.3390/ijerph21040458 PMC11050188 38673369

[cobi70316-bib-0050] Kaur, P. , Saini, J. , Sharma, U. , Duraiswami, R. , Mathew, B. P. , Sreejith, C. , & Kaur, G. (2023). Western Ghats laterite: An architecturally and culturally iconic stone from India with special reference to Goa. Geoheritage, 15(1), Article 38.

[cobi70316-bib-0051] Keith, D. A. , Ferrer‐Paris, J. R. , Nicholson, E. , & Kingsford, R. T. (Eds.). (2020). The IUCN global ecosystem typology 2.0: Descriptive profiles for biomes and ecosystem functional groups. IUCN.

[cobi70316-bib-0052] Keppel, G. , Van Niel, K. P. , Wardell‐Johnson, G. W. , Yates, C. J. , Byrne, M. , Mucina, L. , Schut, A. G. T. , Hopper, S. D. , & Franklin, S. E. (2012). Refugia: Identifying and understanding safe havens for biodiversity under climate change. Global Ecology and Biogeography, 21, 393–404. 10.1111/j.1466-8238.2011.00686.x

[cobi70316-bib-0053] Lajmi, A. , Giri, V. B. , Singh, T. , & Agarwal, I. (2020). Two new species of yellow‐tailed *Hemidactylus* Goldfuss, 1820 (Squamata: Gekkonidae) from rocky outcrops on the Telangana Plateau, India. Zootaxa, 4895(4), 483–504. 10.11646/zootaxa.4895.4.2 33756882

[cobi70316-bib-0054] Lambers, H. , Brundrett, M. C. , Raven, J. A. , & Hopper, S. D. (2010). Plant mineral nutrition in ancient landscapes: High plant species diversity on infertile soils is linked to functional diversity for nutritional strategies. Plant and Soil, 344, 11–31.

[cobi70316-bib-0055] Lèbre, É. , Stringer, M. , Svobodova, K. , Owen, J. R. , Kemp, D. , Côte, C. , Arratia‐Solar, A. , & Valenta, R. K. (2020). The social and environmental complexities of extracting energy transition metals. Nature Communications, 11(1), Article 4823. 10.1038/s41467-020-18661-9 PMC7519138 32973153

[cobi70316-bib-0056] Losekann, C. , & Milanez, B. (2023). Mining disaster in the Doce River: Dilemma between governance and participation. Current Sociology, 71(7), 1255–1273. 10.1177/0011392121105922

[cobi70316-bib-0057] Lullfitz, A. , Pettersen, C. , Reynolds, R. D. , Eades, A. , Dean, A. , Knapp, L. , Woods, E. , Woods, T. , Eades, E. , Yorkshire‐Selby, G. , Woods, S. , Dortch, J. , Guilfoyle, D. , & Hopper, S. D. (2021). The Noongar of south‐western Australia: A case study of long‐term biodiversity conservation in a matrix of old and young landscapes. Biological Journal of the Linnean Society, 133, 432–448.

[cobi70316-bib-0058] Marks, R. A. , Ekwealor, J. T. B. , Artur, M. A. S. , Bondi, L. , Boothby, T. C. , Carmo, O. M. S. , Centeno, D. C. , Coe, K. K. , Dace, H. J. W. , Field, S. , Hutt, A. , Porembski, S. , Thalhammer, A. , Van Der Pas, L. , Wood, A. J. , Alpert, P. , Bartels, D. , Boeynaems, S. , Datar, M. N. , … Rhee, S. Y. (2025). Life on the dry side: A roadmap to understanding desiccation tolerance and accelerating translational applications. Nature Communications, 16(1), Article 3284. 10.1038/s41467-025-58656-y PMC11973199 40189591

[cobi70316-bib-0059] Matos, I. S. , Eller, C. B. , Oliveras, I. , & Mantuano, D. , & Rosado, B. H. (2021). Three eco‐physiological strategies of response to drought maintain the form and function of a tropical montane grassland. Journal of Ecology, 109(1), 327–341. 10.1111/1365-2745.13481

[cobi70316-bib-0060] Merckx, V. S. F. T. , Hendriks, K. P. , Beentjes, K. K. , Mennes, C. B. , Becking, L. E. , Peijnenburg, K. T. C. A. , Afendy, A. , Arumugam, N. , De Boer, H. , Biun, A. , Buang, M. M. , Chen, P.‐P. , Chung, A. Y. C. , Dow, R. , Feijen, F. A. A. , Feijen, H. , Soest, C. F.‐V. , Geml, J. , Geurts, R. , … Schilthuizen, M. (2015). Evolution of endemism on a young tropical mountain. Nature, 524(7565), 347–350. 10.1038/nature14949 26266979

[cobi70316-bib-0061] Millennium Ecosystem Assessment . (2005). Ecosystems and human well‐being: Biodiversity synthesis . World Resources Institute.

[cobi70316-bib-0062] Monteiro, L. , Machado, N. , Martins, E. , Pougy, N. , Verdi, M. , Martinelli, G. , & Loyola, R. (2018). Conservation priorities for the threatened flora of mountaintop grasslands in Brazil. Flora, 238, 234–243.

[cobi70316-bib-0063] Neves, F. S. , Da Silva, P. G. , Solar, R. , Nunes, C. A. , Beirão, M. D. V. , Brant, H. , Castro, F. S , Dáttilo, W. , Guevara, R. , & Fernandes, G. W. (2021). Habitat generalists drive nestedness in a tropical mountaintop insect metacommunity. Biological Journal of the Linnean Society, 133(2), 577–586. 10.1093/biolinnean/blaa059

[cobi70316-bib-0064] Neves, F. S. , Silva, P. G. , Camarota, F. , Nunes, C. A. , Hortal, J. , de Castro, F. S. , Beirão, M. , Ramos, L. , Solar, R. , & Fernandes, G. W. (2025). Complex temporal dynamics of insect metacommunities along a tropical elevational gradient. Ecography, 2025(1), Article e07455. 10.1111/ecog.07455

[cobi70316-bib-0065] Nic Lughadha, E. , Bachman, S. P. , Leão, T. C. C. , Forest, F. , Halley, J. M. , Moat, J. , Acedo, C. , Bacon, K. L. , Brewer, R. F. A. , Gâteblé, G. , Gonçalves, S. C. , Govaerts, R. , Hollingsworth, P. M. , Krisai‐Greilhuber, I. , De Lirio, E. J. , Moore, P. G. P. , Negrão, R. , Onana, J. M. , Rajaovelona, L. R. , … Walker, B. E. (2020). Extinction risk and threats to plants and fungi. Plants, People, Planet, 2(5), 389–408. 10.1002/ppp3.10146

[cobi70316-bib-0066] Nijnens, J. , Behrens, P. , Kraan, O. , Sprecher, B. , & Kleijn, R. (2023). Energy transition will require substantially less mining than the current fossil system. Joule, 7(11), 2408–2413.

[cobi70316-bib-0067] Nyberg, B. , Walsh, S. K. , & Rønsted, N. (2025). The global conservation status of plants growing on cliffs and rocky outcrops. Basic and Applied Ecology, 82, 18–27. 10.1016/j.baae.2024.11.002

[cobi70316-bib-0068] Oliveira, M. F. , Figueredo, C. C. , & Maciel‐Silva, A. S. (2025). Diversity and key organisms in the biocrust of a tropical granite‐gneiss rocky outcrop. Life, 15(5), Article 759. 10.3390/life15050759 PMC12112761 40430187

[cobi70316-bib-0069] Oliveira, M. F. , Santos, P. O. , De Oliveira, R. R. , Figueredo, C. C. , & Maciel‐Silva, A. S. (2025). Encapsulating ecosystem engineers: Alginate beads of biocrusts for restoration efforts. Restoration Ecology, 34(2), Article e70141.

[cobi70316-bib-0070] Oswald, C. B. , Silveira, F. A. O. , Cornelissen, T. , Costa, L. M. , Costa, H. C. , Dagosta, F. C. P. , Domingos, F. M. C. B. , Eterovick, P. C. , Freitas, G. H. S. , Guedes, T. B. , Guerra, T. J. , Goulart, F. F. , Hoffmann, D. , Leite, F. S. F. , Machado, C. G. , Maruyama, P. K. , Oliveira, H. J. , Perini, F. A. , Pezzuti, T. L. , … Magalhães, R. F. (2025). Biogeography and evolution of the vertebrate fauna in *campo rupestre*, a megadiverse Neotropical montane open ecosystem. Biological Journal of the Linnean Society, 145(3), Article blaf032. 10.1093/biolinnean/blaf032

[cobi70316-bib-0071] Ottaviani, G. , & Keppel, G. (2018). Contrasting intraspecific foliar trait responses to stressful conditions of two rhizomatous granite outcrop species at different scales in southwestern Australia. Austral Ecology, 43, 249–256. 10.1111/aec.12571

[cobi70316-bib-0072] Ottaviani, G. , Méndez‐Castro, F. E. , Conti, L. , Zelený, D. , Chytrý, M. , Doležal, J. , Jandová, V. , Altman, J. , & Klimešová, J. (2022). Sticking around: Plant persistence strategies on edaphic islands. Diversity and Distributions, 28(9), 1850–1862.

[cobi70316-bib-0073] Perillo, L. N. , Castro, F. S. , Solar, R. , & Neves, F. S. (2021). Disentangling the effects of latitudinal and elevational gradients on bee, wasp, and ant diversity in an ancient Neotropical mountain range. Journal of Biogeography, 48(7), 1564–1578. 10.1111/jbi.14095

[cobi70316-bib-0074] Perillo, L. N. , Neves, F. D S. , Castro, F. S. D. , & Solar, R. (2023). Neotropical gradients of insect groups in Brazilian mountains. In R. W. Myster (Ed.), Neotropical gradients and their analysis (pp. 309–343). Springer. 10.1007/978-3-031-22848-3_11

[cobi70316-bib-0075] Pillon, Y. , Jaffré, T. , Birnbaum, P. , Bruy, D. , Cluzel, D. , Ducousso, M. , Fogliani, B. , Ibanez, T. , Jourdan, H. , Lagarde, L. , Léopold, A. , Munzinger, J. , Pouteau, R. , Read, J. , & Isnard, S. (2021). Infertile landscapes on an old oceanic island: The biodiversity hotspot of New Caledonia. Biological Journal of the Linnean Society, 133(2), 317–341. 10.1093/biolinnean/blaa146

[cobi70316-bib-0076] Pimenta, M. A. , & Fonseca, A. (2021). To what extent are threatened plant species considered in impact assessment decision‐making? Insights from southeastern Brazil. Environmental Impact Assessment Review, 86, Article 106516.

[cobi70316-bib-0077] Pinceel, T. , Buschke, F. , Weckx, M. , Brendonck, L. , & Vanschoenwinkel, B. (2018). Climate change jeopardizes the persistence of freshwater crustaceans in temporary pools: Linking experimental data with a demographic population model. BMC Ecology, 18(1), Article 2. 10.1186/s12898-018-0158-z PMC5782365 29361977

[cobi70316-bib-0078] Pinceel, T. , Vanschoenwinkel, B. , Brendonck, L. , & Pinceel, J. (2021). An empirical confirmation of diversified bet hedging as a survival strategy in unpredictably varying environments. Ecology, 102(9), Article e03445. 10.1002/ecy.3496 34309020

[cobi70316-bib-0079] Piñeiro, J. M. , Cajade, R. , Hernando, A. B. , Courtis, A. , Ingaramo, M. R. , & Marangoni, F. (2021). The isolated rocky outcrops of northeastern Argentina and their role on the herpetofauna conservation. Anais da Academia Brasileira de Ciências, 93(2), Article e20190932. 10.1590/0001-3765202120190932 34259793

[cobi70316-bib-0080] Poot, P. , Hopper, S. D. , & van Diggelen, J. M. (2012). Exploring rock fissures: Does a specialized root morphology explain endemism on granite outcrops? Annals of Botany, 110(2), 291–300. 10.1093/aob/mcr322 PMC3394634 22238122

[cobi70316-bib-0081] Porembski, S. , Silveira, F. A. O. , Fiedler, P. L. , Watve, A. , Rabarimanarivo, M. , Kouame, F. , & Hopper, S. D. (2016). Worldwide destruction of inselbergs and related rock outcrops threatens a unique ecosystem. Biodiversity and Conservation, 25(13), 2827–2830.

[cobi70316-bib-0082] Reniers, J. , Brendonck, L. , Roberts, J. D. , Verlinden, W. , & Vanschoenwinkel, B. (2015). Environmental harshness shapes life‐history variation in an Australian temporary pool breeding frog: A skeletochronological approach. Oecologia, 178(4), 931–941. 10.1007/s00442-015-3258-x 25694040

[cobi70316-bib-0083] Salerno, P. E. , Ron, S. R. , Señaris, J. C. , Rojas‐Runjaic, F. J. M. , Noonan, B. P. , & Cannatella, D. C. (2012). Ancient tepui summits harbor young rather than old lineages of endemic frogs. Evolution; International Journal of Organic Evolution, 66(10), 3000–3013. 10.1111/j.1558-5646.2012.01666.x 23025594

[cobi70316-bib-0084] Salles, D. M. , Carmo, F. F. D. , & Jacobi, C. M. (2019). Habitat loss challenges the conservation of endemic plants in mining‐targeted Brazilian mountains. Environmental Conservation, 46(2), 140–146.

[cobi70316-bib-0085] Seidou, W. I. , Bondi, L. , Porembski, S. , & Bomisso, E. L. (2025). Desiccation‐tolerant vascular plants: A group of species largely neglected in conservation. Plants, 14(14), Article 2184. 10.3390/plants14142184 PMC12298277 40733421

[cobi70316-bib-0086] Sonter, L. J. , Maron, M. , Bull, J. W. , Giljum, S. , Luckeneder, S. , Maus, V. , Mcdonald‐Madden, E. , Northey, S. A. , Sánchez, L. E. , Valenta, R. , Visconti, P. , Werner, T. T. , & Watson, J. E. M. (2023). How to fuel an energy transition with ecologically responsible mining. Proceedings of the National Academy of Sciences, 120(35), Article e2307006120. 10.1073/pnas.2307006120 PMC10466501 37624732

[cobi70316-bib-0087] Souza‐Filho, P. W. M. , Giannini, T. C. , Jaffé, R. , Giulietti, A. M. , Santos, D. C. , Nascimento, W. R. , Guimarães, J. T. F. , Costa, M. F. , Imperatriz‐ Fonseca, V. L. , & Siqueira, J. O. (2019). Mapping and quantification of ferruginous outcrop savannas in the Brazilian Amazon: A challenge for biodiversity conservation. PLoS ONE, 14(1), Article e0211095. 10.1371/journal.pone.0211095 PMC6336337 30653607

[cobi70316-bib-0088] Stacciarini, J. H. S. , & Gonçalves, R. J. D. A. F. (2025). Energy transition and mining in the Global South. Mercator, 24, Article e24009.

[cobi70316-bib-0089] Thorpe, C. , & Watve, A. (2015). Lateritic plateaus in the northern Western Ghats, India: A review of bauxite mining restoration practices. Journal of Ecological Society, 28(1), 25–44.

[cobi70316-bib-0090] Tibbett, M. (2015). Mining in ecologically sensitive landscapes. CSIRO Publishing.

[cobi70316-bib-0091] Tindano, E. , Lankoandé, B. , Porembski, S. , & Thiombiano, A. (2023). Inselbergs: Potential conservation areas for plant diversity in the face of anthropization. Journal of Phytology, 15, 70–79. 10.25081/jp.2023.v15.8387

[cobi70316-bib-0092] Torres, A. , Zu Ermgassen, S. O. S. E. , Navarro, L. M. , Ferri‐Yanez, F. , Teixeira, F. Z. , Wittkopp, C. , Rosa, I. M. D. , & Liu, J. (2024). Mining threats in high‐level biodiversity conservation policies. Conservation Biology, 38(4), Article e14261. 10.1111/cobi.14261 38571408

[cobi70316-bib-0093] Urban, M. C. (2024). Climate change extinctions. Science, 386(6726), 1123–1128. 10.1126/science.adp4461 39636977

[cobi70316-bib-0094] Vanschoenwinkel, B. , Buschke, F. , & Brendonck, L. (2013). Disturbance regime alters the impact of dispersal on alpha and beta diversity in a temporary pond metacommunity. Ecology, 94(11), 2547–2557. 10.1890/12-1576.1 24400506

[cobi70316-bib-0095] Vanschoenwinkel, B. , de Paula, L. F. A. , Snoeks, J. M. , Van der Stocken, T. , Buschke, F. T. , Porembski, S. , & Silveira, F. A. O. (2025). The ecological and evolutionary dynamics of inselbergs. Biological Reviews, 100(2), 481–507. 10.1111/brv.13150 39352171

[cobi70316-bib-0096] Veldman, J. W. , Overbeck, G. E. , Negreiros, D. , Mahy, G. , Le Stradic, S. , Fernandes, G. W. , Durigan, G. , Buisson, E. , Putz, F. E. , & Bond, W. J. (2015). Where tree planting and forest expansion are bad for biodiversity and ecosystem services. Bioscience, 65(10), 1011–1018.

[cobi70316-bib-0097] Watari, T. , McLellan, B. C. , Giurco, D. , Dominish, E. , Yamasue, E. , & Nansai, K. (2019). Total material requirement for the global energy transition to 2050: A focus on transport and electricity. Resources, Conservation and Recycling, 148, 91–103. 10.1016/j.resconrec.2019.05.015

[cobi70316-bib-0098] Watve, A. , & Chavan, V. (2020). Conceptualising Framework for Local Biodiversity Heritage Sites (LBHS): A Bio‐cultural model for biodiversity conservation in Maharashtra. Asian Biotechnology and Development Review, 22(2‐3), 61–82.

[cobi70316-bib-0099] Wintle, B. A. , Kujala, H. , Whitehead, A. , Cameron, A. , Veloz, S. , Kukkala, A. , Moilanen, A. , Gordon, A. , Lentini, P. E. , Cadenhead, N. C. R. , & Bekessy, S. A. (2019). Global synthesis of conservation studies reveals the importance of small habitat patches for biodiversity. Proceedings of the National Academy of Sciences, 116(3), 909–914. 10.1073/pnas.1813051115 PMC6338828 30530660

[cobi70316-bib-0100] Zeppelini, D. , Oliveira, J. V. L. , de Lima, E. C. A. , Brito, R. A. , Ferreira, A. S. , Stievano, L. C. , Brito, N. P. , Oliveira‐Neto, M. A. , & Lopes, B. C. (2022). Hotspot in ferruginous rock may have serious implications in Brazilian conservation policy. Scientific Reports, 12(1), Article 14871. 10.1038/s41598-022-18798-1 PMC9437091 36050352

