# Supplementary material for: Scientists’ warning on the global destruction of rock outcrop ecosystems
Source: Conserv Biol. 2026 May 9;40(5):e70316. doi: 10.1111/cobi.70316 (PMC13613563; doi:10.1111/cobi.70316)
Supplement: Supplementary file 1 [file COBI-40-e70316-s001.docx]

**Supplementary Material**

**Table S1**. Key ecosystem services and values associated with rock outcrop ecosystems worldwide. Services and values are grouped in categories such as provisioning (P), regulating (R), supporting (S), or cultural (C).

| **Category** | **Ecosystem services and values** | **Rock outcrops** | **References** |
| --- | --- | --- | --- |
| P | Water recharge and provision | Quartzitic and ironstone campos rupestres (Brazil); granite outcrops (Brazil); karst terrains (worldwide); laterite (Northern Western Ghats, India); campos de altitude | Lehmann et al. 2022; Goldscheider et al. 2019; [Buono et al. 2015](https://www.scienceopen.com/document_file/fa1ce4b0-e731-47d9-8677-b10b14ef4821/ScienceOpen/Vol%2028_8-24.pdf); Ribeiro & Freitas 2010; Silva et al. 2026 |
| P | Plant diversity storage and trade of ornamental plants | Campos rupestres and campos de altitude (Brazil) | Neves et al. 2016;  [Matos et al. 2021](https://besjournals.onlinelibrary.wiley.com/doi/full/10.1111/1365-2745.13481); Ribeiro & Freitas 2010 |
| R | Carbon storage and nutrient cycling | Campos rupestres and granite outcrops (Brazil); karst terrains (worldwide) | Silva et al. 2026; Couto et al. 2025; Goldscheider et al. 2019 |
| R | Erosion control and soil stabilization | Karst terrains (worldwide);  ironstone geosystems (Brazil) | Goldscheider et al. 2019; [Gagen et al. 2019](https://www.sciencedirect.com/science/article/abs/pii/S0169136817309587) |
| R | Climate regulation (microclimatic buffering) | Granite outcrops (southwestern Australia) | Schut et al. 2014; Ottaviani et al. 2016 |
| S | Habitat provision and biodiversity refugia | Tepuis (Northern South America); granite outcrops (southeastern Brazil; southwestern Australia and French Guiana); campos rupestres (Brazil); ironstone geosystems (Brazil and Australia); karst terrains (worldwide); campos de altitude (Brazil) | [Rull et al. 2019;](https://www.sciencedirect.com/science/chapter/edited-volume/abs/pii/B978012815591200001X?via%3Dihub) de Paula et al. 2015; Ottaviani et al. 2016; Keppel et al. 2017; Henneron et al. 2019; Silveira et al. 2016; Zeppelini et al. 2022; Gibson et al. 2015; Goldscheider et al. 2019; [Kessous (2026)](https://conbio.onlinelibrary.wiley.com/doi/full/10.1111/csp2.70238) |
| S | Pollination services | Campos rupestres and campos de altitude (Brazil) | Cardoso et al. 2026; Ribeiro & Freitas 2010 |
| C | Cultural, spiritual, and aesthetic values | Various rock outcrops (worldwide); karst terrains (worldwide); granite outcrops (Australia); campos de altitude and campos rupestres (Brazil) | Goldscheider et al. 2019; Day & Urich 2000; Lullfitz et al. 2021; Neves et al. 2016 |
| C | Tourism, recreation, and education | Tower karsts (China) and karst areas (Brazil); campos rupestres and ironstone geosystems (Brazil); granite outcrops (Brazil) | Day & Urich 2000; Ruchkys & Auler 2025  Neves et al. 2016; de Paula et al. 2026 |

**References**

Buono, J., Thomas, R., Kulkarni, H., Mahamuni, K., Karandikar, M., Ghate, K., ... & More, A. (2015). Ecohydrologic description of springs in the North Western Ghats, Maharashtra. *J. Ecol. Soc.*, **28**, 8–24.

Cardoso, J. C., Trevizan, R., Maruyama, P. K., Caetano, A. P., Gonçalves, R. V., Antonini, Y., & Oliveira, P. E. (2026). Pollination and plant reproduction in the Cerrado, the world's most biodiverse savanna. *Biological Reviews*, **101**(1), 74–105.

Couto, D. R., Francisco, T. M., de Paula, L. F. A., Paula, R. R., & Nascimento, M. T. (2025). Woody vegetation on tropical inselbergs: floristic-structural characterization and aboveground carbon storage. *Journal of Mountain Science*, **22**(5), 1517–1534.

Day, M., & Urich, P. (2000). An assessment of protected karst landscapes in Southeast Asia. *Cave and Karst Science*, **27**(2), 61–70.

de Paula, L. F., Negreiros, D., Azevedo, L. O., Fernandes, R. L., Stehmann, J. R., & Silveira, F. A. (2015). Functional ecology as a missing link for conservation of a resource-limited flora in the Atlantic forest. *Biodiversity and Conservation*, **24**(9), 2239-2253.

de Paula, L. F. A., Francisco, T. M., Fraga, C. N., Deccache, L. S. J., Forzza, R. C., & Couto, D. R. (2026). The flora islanded by forests: a synthesis of the diversity, ecology, and conservation of inselbergs in the Atlantic Forest. *Boletim do Museu de Biologia Prof. Mello Leitão – Série INMA*, **in press**.<https://doi.org/10.70525/2318-9444.2025.30>

Gagen, E. J., Levett, A., Paz, A., Gastauer, M., Caldeira, C. F., da Silva Valadares, R. B., ... & Southam, G. (2019). Biogeochemical processes in canga ecosystems: Armoring of iron ore against erosion and importance in iron duricrust restoration in Brazil. *Ore Geology Reviews*, **107**, 573–586.

Gibson, N., Coates, D., van Leeuwen, S., & Yates, C. (2015). Hot, dry and ancient: banded iron formations of Western Australia. In F. F. Carmo & L. H. Kamino (Eds.), *Geossistemas ferruginosos do Brasil: áreas prioritárias para conservação da diversidade geológica e biológica, patrimônio cultural e serviços ambientais* (pp. 361–391). Belo Horizonte, Brazil: Instituto Prístino.

Goldscheider, N. (2019). A holistic approach to groundwater protection and ecosystem services in karst terrains. *Carbonates and Evaporites*, **34**(4), 1241–1249.

Henneron, L., Sarthou, C., de Massary, J. C., & Ponge, J. F. (2019). Habitat diversity associated to island size and environmental filtering control the species richness of rock‐savanna plants in neotropical inselbergs. *Ecography*, **42**(9), 1536–1547.

Keppel, G., Robinson, T. P., Wardell-Johnson, G. W., Yates, C. J., Van Niel, K. P., Byrne, M., & Schut, A. G. (2017). A low-altitude mountain range as an important refugium for two narrow endemics in the Southwest Australian Floristic Region biodiversity hotspot. *Annals of Botany*, **119**(2), 289–300.

Kessous, I. M. (2023). Campos de altitude from Brazil: neglected biodiversity treasures under threat. *Conservation Science and Practice*, **5**, e70238.

Lehmann, J. R., Prior, M. D., de Paula, L. F. A., Azevedo, L., Porembski, S., Buttschardt, T. K., & Solar, R. (2022). Using drone imagery to upscale estimates of water capacity in tank bromeliads on steep neotropical inselbergs. *Austral Ecology*, **47**(2), 196–202.

Lullfitz, A., Pettersen, C., Reynolds, R., Eades, A., Dean, A., Knapp, L., … & Hopper, S. D. (2021). The Noongar of south-western Australia: a case study of long-term biodiversity conservation in a matrix of old and young landscapes. *Biological Journal of the Linnean Society*, **133**, 432–448.

Matos, I. S., Eller, C. B., Oliveras, I., Mantuano, D., & Rosado, B. H. (2021). Three eco-physiological strategies of response to drought maintain the form and function of a tropical montane grassland. *Journal of Ecology*, **109**(1), 327–341.

Neves, A. C. D. O., Barbieri, A. F., Pacheco, A. A., Resende, F. D. M., Braga, R. F., Azevedo, A. A., & Fernandes, G. W. (2016). The human dimension in the Espinhaço mountains: Land conversion and ecosystem services. In G. W. Fernandes (Ed.), *Ecology and Conservation of Mountaintop grasslands in Brazil* (pp. 501–530). Springer International Publishing.

Ottaviani, G., Marcantonio, M., & Mucina, L. (2016). Soil depth shapes plant functional diversity in granite outcrops vegetation of Southwestern Australia. *Plant Ecology & Diversity*, **9**(3), 263–276.

Ribeiro, K., & Freitas, L. (2010). Impactos potenciais das alterações no Código Florestal sobre a vegetação de campos rupestres e campos de altitude. *Biota Neotropica*, **10**, 239–246.

Ruchkys, Ú. D. A., & Auler, A. S. (2025). Geoparks in Brazilian karst areas: institutional scenarios and multilevel governance strategies. *Environmental Earth Sciences*, **84**(15), 437.

Rull, V., Huber, O., Vegas-Vilarrúbia, T., & Señaris, C. (2019). Definition and characterization of the Pantepui biogeographical province. In *Biodiversity of Pantepui* (pp. 3–32). Academic Press.

Schut, A. G., Wardell-Johnson, G. W., Yates, C. J., Keppel, G., Baran, I., Franklin, S. E., ... & Byrne, M. (2014). Rapid characterisation of vegetation structure to predict refugia and climate change impacts across a global biodiversity hotspot. *PLoS ONE*, **9**(1), e82778.

Silva, K. F., Meyer, L., Resende, F. M., Silveira, F. A., & Fernandes, G. W. (2026). Potential mining impacts on ecosystem services and biodiversity on Brazil's quartz and iron mountains. *Science of The Total Environment*, **1016**, 181479.

Silveira, F. A., Negreiros, D., Barbosa, N. P., Buisson, E., Carmo, F. F., Carstensen, D. W., ... & Lambers, H. (2016). Ecology and evolution of plant diversity in the endangered campo rupestre: a neglected conservation priority. *Plant and Soil*, **403**(1), 129–152.

Zeppelini, D., Oliveira, J. V. L., de Lima, E. C. A., Brito, R. A., Ferreira, A. S., Stievano, L. C., ... & Lopes, B. C. (2022). Hotspot in ferruginous rock may have serious implications in Brazilian conservation policy. *Scientific Reports*, **12**(1), 14871.
